# A Hidden Portrait by Edgar Degas

**DOI:** 10.1038/srep29594

**Published:** 2016-08-04

**Authors:** David Thurrowgood, David Paterson, Martin D. de Jonge, Robin Kirkham, Saul Thurrowgood, Daryl L. Howard

**Affiliations:** 1 National Gallery of Victoria, Melbourne, Victoria, Australia; 2 Australian Synchrotron, Clayton, Victoria, Australia; 3 The Commonwealth Scientific and Industrial Research Organisation, Clayton, Victoria, Australia; 4 Queensland Brain Institute, University of Queensland, Brisbane, Queensland, Australia

## Abstract

The preservation and understanding of cultural heritage depends increasingly on in-depth chemical studies. Rapid technological advances are forging connections between scientists and arts communities, enabling revolutionary new techniques for non-invasive technical study of culturally significant, highly prized artworks. We have applied a non-invasive, rapid, high definition X-ray fluorescence (XRF) elemental mapping technique to a French Impressionist painting using a synchrotron radiation source, and show how this technology can advance scholarly art interpretation and preservation. We have obtained detailed technical understanding of a painting which could not be resolved by conventional techniques. Here we show 31.6 megapixel scanning XRF derived elemental maps and report a novel image processing methodology utilising these maps to produce a false colour representation of a “hidden” portrait by Edgar Degas. This work provides a cohesive methodology for both imaging and understanding the chemical composition of artworks, and enables scholarly understandings of cultural heritage, many of which have eluded conventional technologies. We anticipate that the outcome from this work will encourage the reassessment of some of the world’s great art treasures.

Preserving and interpreting the world’s material cultural heritage requires increasingly sophisticated understandings of chemical composition, environmental history, and deterioration mechanisms^1^. Knowledge and understanding of historic materials has conventionally required the removal of samples which are subjected to analytical techniques, and the process frequently alters or destroys the specimen. Even sub-millimetre sampling “damage” to works of substantial cultural heritage can be unacceptable for highly valued objects. In art examination it is highly desirable that materials can be identified without sampling, and without change to the material being studied. Conventional analytical techniques have given inconclusive outcomes, in particular where the area of interest is obscured by an upper layer^2^,^3^.

Concealed paintings, early compositions that have been hidden by subsequent work, are important insights into artworks and artists. They can reveal the evolution of an artist’s technique and can prove invaluable to the attribution of a work^4^,^5^. Conventional X-radiography of paintings has been undertaken since 1896, and has been heavily relied upon in the understanding of paintings^6^. X-ray absorption is mainly provided by the heavy metal components of pigments used, and the technique provides minimal quantitative or specific elemental identification information. The interpretation of X-radiography images is a highly subjective process. In recent years considerable effort has been expended into developing large-area non-invasive examination techniques of artworks and archaeometric study of objects to fulfil a growing need to accurately understand the elemental and molecular composition of artworks^7^,^8^,^9^,^10^,^11^,^12^,^13^,^14^,^15^,^16^. This new analytical information has become critical in attribution and degradation studies and art historical assessments and is used to direct the practices of art conservators as they seek to implement new preservation strategies.

It has been demonstrated with the X-ray fluorescence (XRF) technique that metallic elements from pigments in an underpainting can be detected and resolved with sufficient sensitivity to enable reconstruction of concealed paint layers^2^,^4^,^5^,^7^,^10^,^17^. The first major synchrotron study, which revealed a woman’s head under the Van Gogh painting *Patch of Grass* required extended examination time (~2 days, 2 second per pixel dwell time), and produced modest resolution 0.5 mm over an area of only 175 × 175 mm^2 ^2. This showed the power of scanning XRF, but also highlighted what had been the traditional limitation of slow pixel acquisition rates, which often resulted in compromises to the overall scan size and/or spatial resolution. In recent years the development of rapid scanning XRF methods^8^,^19^,^20^ with millisecond analysis times have dramatically improved data collection rates, enabling the potential to measure a significant portion of a painting at spatial resolutions on the order of the size of a paint brush bristle^10^,^21^.

Here we demonstrate a non-invasive examination of a concealed painting, and deliver new art historical observations, made possible with high-definition scanning XRF analysis^4^,^10^,^21^. We illustrate this by investigating a previously difficult to interpret^22^ portrait by Edgar Degas (1834–1917), one of the greatest French painters of the 19th century and a founding member of Impressionism. We have examined his painting *Portrait of a Woman* (*Portrait de Femme*, oil on canvas, 463 × 382 mm^2^, painted circa 1876–1880) from the collection of the National Gallery of Victoria, Australia (Fig. 1a).

## Results

### Conventional imaging

**Figure 1:**
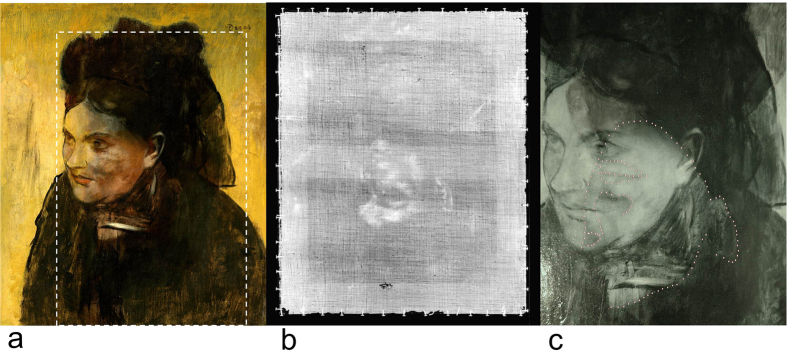
*Portrait of a Woman*. Edgar Degas, French, 1834–1917, Portrait of a Woman (Portrait de Femme), c. 1876–80, oil on canvas, 46.3 × 38.2 cm, National Gallery of Victoria, Melbourne, Felton Bequest, 1937. (**a**) Visible light image. The boxed region highlights the XRF scan area. (**b**) X-radiograph. The obscured portrait is rotated 180 degrees relative to the upper portrait. The face and ear of the obscured sitter are the primary source of contrast. (**c**) Reflected infrared image (detail). A partial outline of the obscured sitter’s face is indicated with a dotted line. The extensive use of highly infrared-absorbing black paint in the final composition provides a limited view of the underlying figure.

*Portrait of a Woman* by Edgar Degas (Fig. 1a) has historically been known to have a concealed figure, and the work has been criticised since at least 1922 for the gradually increasing outline of the underpainting^22^. Degas painted directly on the underlying portrait with no intermediate ground paint layer using exceptionally thin paint layers, thus little pigment is present to provide hiding power. The hiding power of paint layers often decreases as oil paintings age. The index of refraction of the natural oil medium has a tendency to increase over time, thus the difference between the pigment’s and the oil’s indices of refraction become smaller, leading to less light scattering at the oil-pigment interface and therefore yielding lower opacity^23^. The gradual increase in transparency of pigments such as emerald green (Cu(C_2_H_3_O_2_)_2_·3Cu(AsO_2_)_2_)^24^ and lead-based pigments^25^ has been observed and studied, with metal soap formation considered to play a major role in the process^24^,^26^.

The identity of the woman in the black dress and bonnet is currently unknown. In the visible light image (Fig. 1a), it can be observed that the underlying portrait runs in the opposite orientation to the upper composition. The shoulders of the hidden portrait are the source of the diagonal lines radiating from the present sitter’s bonnet to the top corners of the painting. The sitter’s face appears discoloured as the impression of the hidden composition shows through.

An X-radiographic image (Fig. 1b) and a reflected infrared image (Fig. 1c) of the painting represent the limits of conventional practice for imaging the work^27^. The X-radiographic image indicates that the underlying portrait is a young woman in three-quarter view. The main source of contrast is provided by the face and ear. Infrared imaging is sensitive to carbon-based pigments and is often used to reveal underdrawings which may consist of graphite or charcoal for example^28^,^29^. In the reflected infrared image, it is apparent that the black paint used in the garment of the upper painting has high opacity to infrared radiation (Fig. 1c). This is indicative of a carbon-based pigment such as carbon black, and its extensive use in the present portrait provides limited views to the underlying work. Portions of the underlying figure’s face are observed where it is overlapped by the present sitter’s face. Overall the underpainting cannot be resolved as more than a faintly outlined female figure by conventional techniques, and it has long been considered to be indecipherable, to the disappointment of academics studying the artwork^22^.

### High-definition XRF mapping

**Figure 2:**
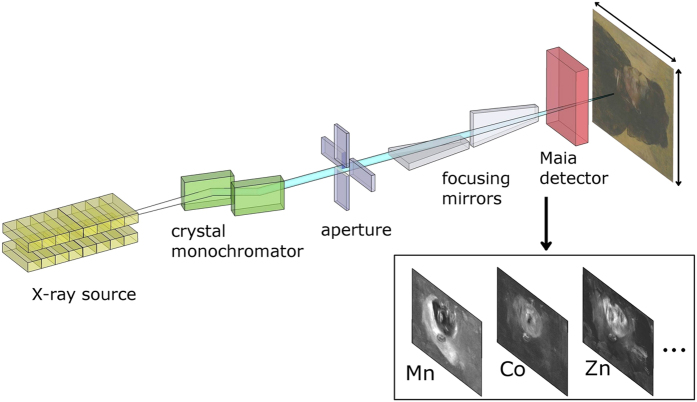
Simplified schematic of the synchrotron-based scanning X-ray fluorescence microscope. A monochromatic undulator-based X-ray source is focused by Kirkpatrick-Baez mirrors. The focused beam passes through an aperture in the Maia detector onto the raster-scanned sample. The X-ray photon events are detected in a backscatter geometry and analysed to produce elemental and scatter maps. (Edgar Degas, French, 1834–1917, Portrait of a Woman (Portrait de Femme), c. 1876–80, oil on canvas, 46.3 × 38.2 cm, National Gallery of Victoria, Melbourne, Felton Bequest, 1937).

**Figure 3:**
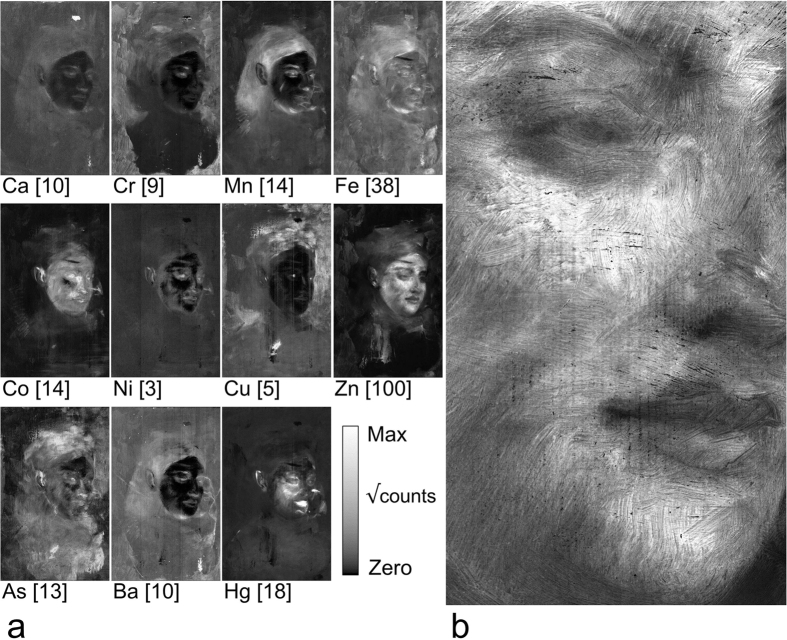
High-definition 31.6 megapixel X-ray fluorescence elemental maps of *Portrait of a Woman*. (**a**) Eleven elemental maps providing an overview of the construction of the painting (426 × 267 mm^2^ scan). The maps have been downsized by averaging over 4×4 pixels and displayed as the square root of the elemental counts with the threshold value displayed in square brackets (e.g., Zn has a maximum display threshold of 100 counts, corresponding to 10^4^ photons per pixel). (**b**) Detail of zinc map, also in square-root counts. The fine brush work of the hidden sitter is clearly revealed. Image size shown is approximately 118×66 mm^2^ (~2.2 Mpixel). (Edgar Degas, French, 1834–1917, Portrait of a Woman (Portrait de femme) c. 1876–80, oil on canvas, 46.3 × 38.2 cm, National Gallery of Victoria, Melbourne, Felton Bequest, 1937).

The use of the Maia 384 detector array^30^ at the X-ray Fluorescence Microscopy (XFM) beamline^31^ has enabled the rapid collection of high-definition synchrotron XRF data over areas spanning tens of dm^2^ (Fig. 2). The 31.6 megapixel scanning XRF elemental maps obtained from *Portrait of a Woman* are presented in Fig. 3. Under the experimental conditions, relevant detectable elements range from K-edge excitations of *Z* = 20–33 (Ca to As) and L-edge excitations of *Z* = 50–80 (Sn to Hg).

XRF imaging can be used to deduce pigment use based on the elements observed within the context of the painting. However it cannot be used to unequivocally identify pigments. Pigment identification can be further supported when different elements are highly co-located. Co-located elements could indicate a pigment intrinsically containing different metals or a mixture of pigments used by the artist to achieve the desired colour. For instance, Fe and Mn are co-located in the hidden sitter’s hair (Fig. 3a), strongly suggesting the use of the brown pigment umber, a fine-grained rock consisting of manganese oxides and hydroxides (5–20% composition) with iron oxyhydroxides (~45–70%)^32^. The Fe:Mn atomic ratio of 6:1 determined from the XRF data is consistent with the composition of umber and its use in the hair.

The As and Cu maps suggest a possible headdress or adornment was attempted on the hidden sitter (Fig. 3a). Cu and As are often associated with green pigments belonging to the copper arsenite group^32^. A historic pigment commonly associated with the copper arsenites is Scheele’s green, which is considered to be a mixture of several components of varying composition, (e.g., copper diarsenite (2CuO·As_2_O_3_·H_2_O), copper metaarsenite (CuO·As_2_O_3_), copper arsenate (Cu(AsO_2_)_2_), etc.)^32^. In regions with high correlation of As and Cu, we find the atomic ratio of As:Cu as 2:1, consistent with the use of copper arsenite. Arsenic is also present in the hair and bonnet of the upper composition; however these areas are relatively free of Cu, barring a region of repair, suggesting the presence of another arsenic pigment. Potential arsenic pigments are likely arsenic-sulphur based such as realgar (As_2_S_2_, orange-red), pararealgar (As_4_S_4_, red-orange) or orpiment (As_2_S_3_, yellow to greenish-yellow), however the relatively low energy sulphur fluorescence (~2.3 keV) is below the low energy limit of the fluorescence detector to aid this line of reasoning.

The hidden sitter’s face consists of several elements. The Zn map provides the best overview of the face and showcases Degas’ brushwork (Fig. 3b). Here Zn would most likely be in the form of zinc white pigment (ZnO), which came into widespread use after 1845^32^. Zinc appears to be the most thickly applied element detected on the face, and it also present in the ear and hair of the hidden sitter. Similar to the Zn map, Co defines the hidden sitter’s face and ear, and it is also present in the hidden sitter’s garment. Cobalt is probably present as a blue pigment, which is useful in defining flesh tones, with examples being cobalt blue (CoAl_2_O_4_) or smalt (Co-doped alkali glass). Mercury is predominant in the facial area and would most likely correspond to the red pigment vermilion (HgS), which would contribute to a pink flesh tone. It is primarily used on the lips, face and ears of the hidden portrait. Cobalt is also present on the lips of the present portrait. Iron is also present in areas of the face that are free of Mn, suggesting another pigment besides umber as was postulated for the hair. Hematite (Fe_2_O_3_) and goethite (FeO(OH)) are plausible pigments for use in the creation of flesh tones as they have the ability to generate red and yellow hues.

The background of the painting is defined by chromium, which is also present in some features of the hidden sitter’s face such as the eyes. Based on the background colour, several possibilities exist such as chrome yellow (PbCrO_4_, PbCrO_4_·*x*PbSO_4_ or PbCrO_4_·*x*PbO). Chrome yellow has a tendency to darken with time and its degradation process with respect to the works of Van Gogh has been studied in detail^33^,^34^,^35^,^36^. The Zn:Cr ratio varies over the background, ranging from approximately 1:1 above the hidden sitter’s head to 5:1 on the left and right sides of the painting. Based on the 1:1 Zn:Cr ratio, another possibility is the use of zinc yellow (K_2_O·4ZnCrO_4_·3H_2_O)^32^ or the green pigment viridian (Cr_2_O_3_·2H_2_O) augmented with a Zn-containing pigment. Zinc yellow is also sensitive to degradation, with George Seurat’s masterpiece *A Sunday on La Grande Jatte* displaying the effect^37^,^38^.

The overall counts observed for Ni is low, suggesting Ni is present in low concentrations or in lower paint layers where its fluorescence would be more attenuated. At the cost of lower spatial resolution, an improved signal to noise was achieved by averaging the map over 4 × 4 pixels, which is an advantage of high definition mapping when counting statistics are low. The Ni map shows a general Ni distribution throughout the painting, and it appears that Ni is predominant in areas of the hidden sitter’s face. The rather uniform distribution throughout the remainder of the painting may suggest that Ni is present in the ground layer. Based on the rather low counts observed for Ni, it is unlikely a Ni-based pigment. Nickel is often present as an impurity in many pigments, with lead white or cobalt-based pigments being common examples^39^.

The hidden sitter’s garment forms an outline showing through the upper composition in the visible light image of the painting (Fig. 1a), and it is found to consist primarily of Mn, Co and Hg. These elements do not appear to cover the whole of the hidden garment, thus it may also consist of, for example, low *Z* inorganic pigments, carbon-based black or other organic dye-based pigments which are undetectable by XRF.

The black painted areas of the upper artwork have low concentrations of Ca (Fig. 3a). Thus the black pigment used is unlikely to belong to the cokes family of black pigments, such as bone black^32^. Bone black contains approximately 84 wt% Ca_3_(PO_4_)_2_^40^, thus Ca fluorescence would be readily observed from the painting surface if bone black had been used. The upper portrait’s pigment composition is more likely to belong to the flame carbons family of blacks, such as lamp black, with the carbon source possibly from a hydrocarbon precursor^32^.

The Ba distribution image is particularly useful in identifying the location of the upper portrait relative to the earlier composition. Barium is routinely observed in art as barium sulphate (BaSO_4_) and was in common use for preparation of commercial canvas grounds and used as a low-cost filler material or hue alteration in commercial pigment mixtures, including zinc white and viridian^32^.

### X-ray scatter maps

**Figure 4:**
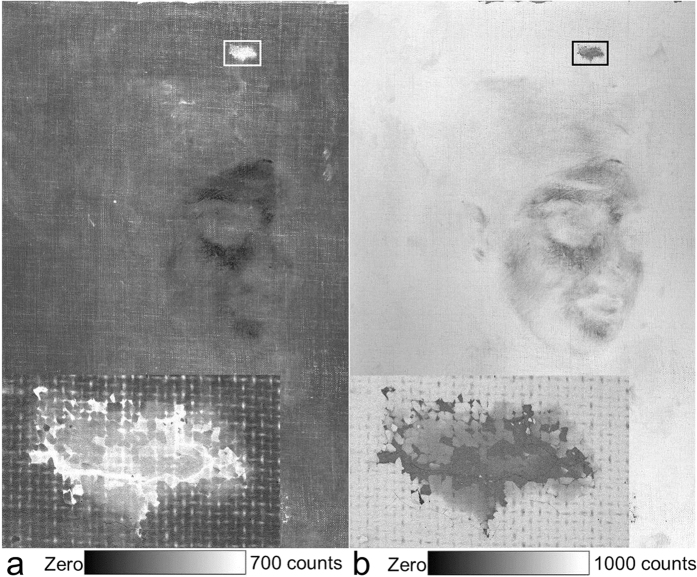
X-ray scatter maps. (**a**) Inelastic and (**b**) elastic scatter. The boxed areas are shown inset for greater detail, and reveal an approximate 21 × 13 mm^2^ puncture damage, previously restored by filling and overpainting, and not readily evident by visible light examination. Maps displayed in a linear count scale.

The X-ray scatter maps (Fig. 4) provide further complementary information to the elemental maps. The inelastic (Compton) scatter is sensitive to the lighter elements and thus enables imaging of dense organic components such as the canvas (Fig. 4a)^10^. In contrast, the elastic (Rayleigh) scatter is more sensitive to the heavier elements such as lead and mercury (Fig. 4b). Here the elastic scatter provides an image of the ground layer and for example, the lead white paint brush stroke running across the hidden sitter’s forehead, and is complementary to the inelastic scatter map. Earlier damage and restoration is clearly identifiable in the scatter maps and is highlighted in Fig. 4. A negative image of the hidden sitter’s face is observed in both scatter maps. We attribute this primarily to the heavy application of zinc-based paint in this area (Fig. 3), which would attenuate the incident X-ray beam and then further attenuate (self-absorb) the scattered X-rays from the underlying ground layer and canvas. Overall these attenuation effects would yield lower sensitivity to materials below the zinc layer.

### Pb Raman Imaging

X-ray Raman scattering is an inelastic scattering of X-rays from core electrons. It is normally a weak process, but can become considerably stronger through a resonance effect if the incident photon energy is immediately below the absorption edge of a matrix material^41^. The incident beam excitation energy, 12.6 keV, was chosen ~0.4 keV below the Pb L_3_ edge to minimize the Raman scattering signal from the Pb-rich pigments and ground layer. Another consideration in the choice of energy was to remain above the Hg L_3_ absorption edge at 12.284 keV and keep the incident energy high enough to minimise the inelastic scatter tail from interfering with fluorescence lines and thereby limiting sensitivity. We have previously used the 12.6 keV incident beam energy to successfully image a painting wholly covered in lead white paint^10^. The Maia 384A detector’s low energy sensitivity cutoff is approximately 4 keV, so (surface) Pb detection via the low energy Pb M fluorescence lines (~2.3 keV) was not possible. In this work we found that Raman scattering could be detected and used for imaging Pb.

**Figure 5:**
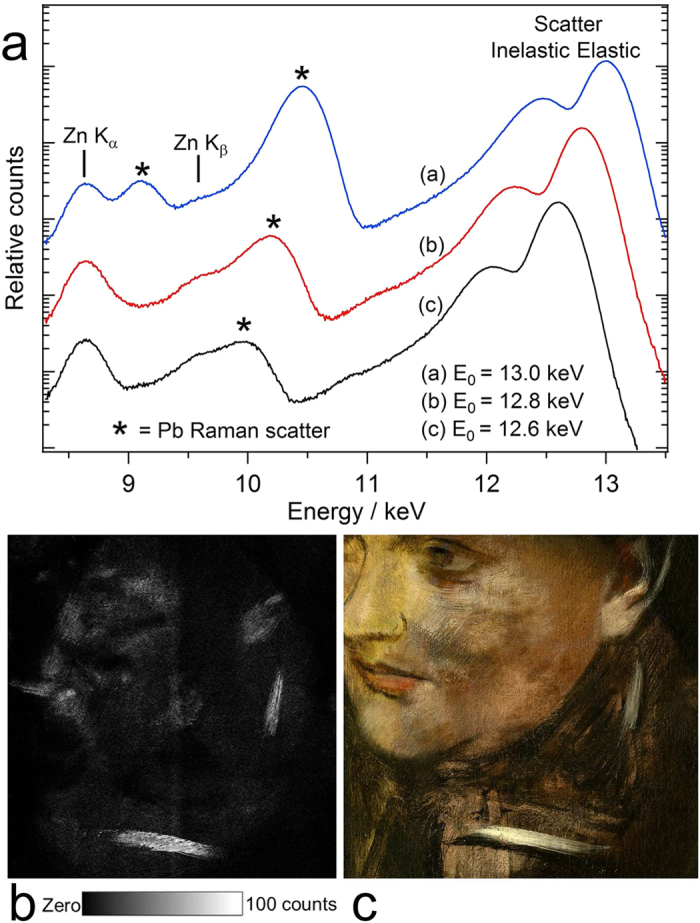
Pb Raman imaging. (**a**) X-ray spectra obtained from a lead white-containing paint sample demonstrating Raman scattering for three excitation energies below the Pb L_3_ absorption edge (13.035 keV). The spectra have been offset by factors of 10 for clarity. (**b**) Pb Raman scatter map (detail) indicates areas of lead-based paint application to the painting. Map displayed in a linear count scale. (**c**) Detail of *Portrait of a Woman* shows that the white brush strokes yield the strongest Pb Raman signal. The low intensity of the Raman scatter at 12.6 keV excitation energy renders surface Pb most sensitive to detection, as scatter from below the surface would be attenuated by the overlying high density paint layers. (Edgar Degas, French, 1834–1917, Portrait of a Woman (Portrait de Femme), (**c**). 1876–80, oil on canvas, 46.3 × 38.2 cm, National Gallery of Victoria, Melbourne, Felton Bequest, 1937).

To demonstrate the Raman scattering effect below the Pb L_3_ edge (13.035 keV), Fig. 5a shows logarithmic plots of three spectra of a paint sample containing lead white (basic lead carbonate, 2PbCO_3_·Pb(OH)_2_) obtained at 12.6, 12.8 and 13.0 keV. Zn fluorescence, originating from the canvas preparation, is also indicated in the plot. The ratio of the integrated intensity of the most intense Raman scattering band to the elastic (Rayleigh) scatter I_Raman_/I_elastic_ = 0.55 at 13.0 keV, and I_Raman_/I_elastic_ = 0.034 at 12.6 keV, illustrating the rapid intensity drop of the Raman signal as a function of energy below the Pb L_3_ edge. The change in elastic scatter intensity was negligible over this incident energy range.

Due to its relatively low intensity at 12.6 keV excitation energy, the Pb Raman scattering was best detected in areas of the portrait containing surface applications of Pb-based paint, in particular the white brush strokes below and to the right of the sitter’s face (Fig. 5b,c). A relatively low Pb Raman signal was observed for the Cr-containing background of the painting, which may support that a non-Pb based Cr-containing pigment such as zinc yellow or viridian was used rather than Pb-based chrome yellow (*vide supra*).

The Raman signal is likely not practical to use as a reliable imaging method for paintings given their highly variable nature. However it does highlight that the choice of excitation energy is an important experimental consideration when working immediately below an absorption edge of any painting component.

### Colour Reconstruction

Elemental maps enable false colour reconstruction of concealed artworks, which provide insight to the colour palette of the artist. Previous researchers^2^,^42^ have attributed a false colour to their elemental maps, and were able to create a plausible colour representation of the underpainting. For instance, with the Van Gogh painting *Patch of Grass* the false colour effect was achieved by manually overlaying two elemental maps with manually assigned colour and transparency^2^.

**Figure 6:**
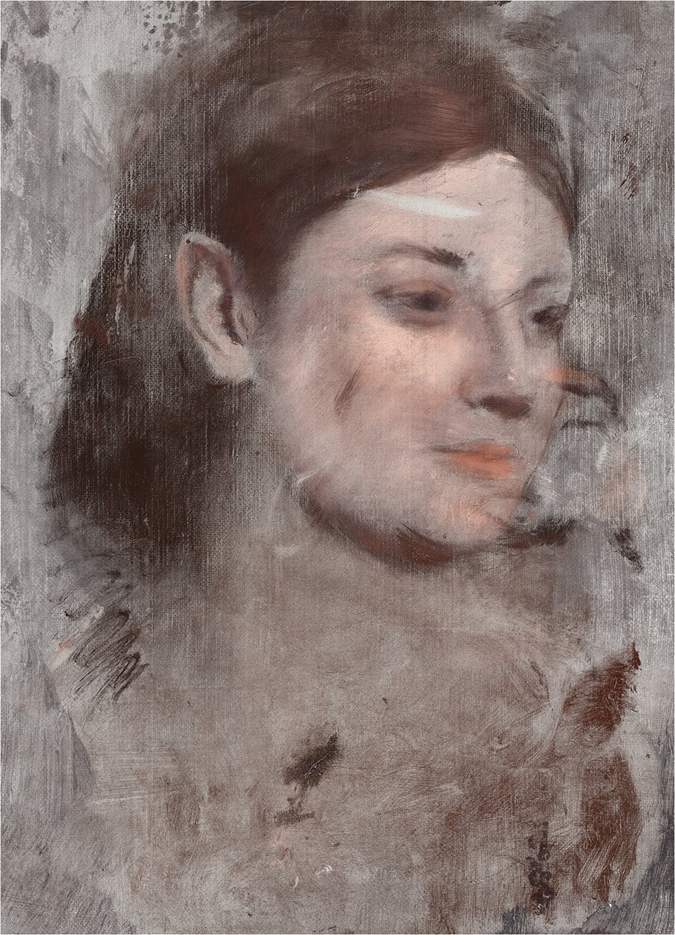
The hidden portrait of Emma Dobigny. False colour reconstruction of Degas’ hidden portrait (detail). The image was created from the X-ray fluorescence microscopy elemental maps. (Edgar Degas, French, 1834–1917, Portrait of a Woman (Portrait de femme) c. 1876–80, oil on canvas, 46.3 × 38.2 cm, National Gallery of Victoria, Melbourne, Felton Bequest, 1937).

A false colour image of the underlying painting from *Portrait of a Woman* was made using a methodology for layering multiple elemental maps. It was generated using custom-written software capable of merging the high resolution, high dynamic range elemental images manually assigned with colours most likely associated with each element (e.g., red for Hg, blue for Co). The colour is chosen based on published examples of the typical colour of a pigment. Pigment colour is not a standardized value, and varies considerably in natural pigments^43^. The resulting false colour image (Fig. 6) is a plausible representation of the artist’s work from the period^44^,^45^, and we have presented it to emphasise the underlying image.

### The Hidden Portrait

Based on the observed XRF elemental maps, we propose that the revealed underpainting is a previously unknown portrait of the model Emma Dobigny. Dobigny, whose real name was Marie Emma Thuilleux, modelled for Degas between 1869–1870 and is reported as a favourite model of Degas and other French artists of the period^44^. We observe strong resemblance between the revealed underpainting and several of Degas’ portraits of Emma Dobigny. Literature suggests that Degas had a special fondness for Emma Dobigny^44^ which may account for the otherwise unfinished or unsatisfactory painting being retained by Degas.

## Discussion

The high-definition XRF maps have recorded the construction of the painting and its condition. Regions of change, alteration or inconsistent pigment presence are immediately evident from the elemental and scatter maps. Degas’ transformation of palette and technique is clearly documented with the years spanning the hidden portrait of Emma Dobigny (c. 1869) and the unknown sitter (c. 1876–1880). The more conventional, thicker image layer of the underpainting is juxtaposed strikingly to the thinly applied later painting, and his change in palette provides exceptional elemental contrast. These maps open up new possibilities for detailing the condition of an artwork at a point in time, and provide a method for non-invasively identifying artist technique in previously unimaginable detail.

The high definition scanning XRF technique allows the plausible attribution of pigments to regions, and is a significant advance on conventional X-radiography which provides only pigment density for a relatively limited number of (mostly heavy) elements. While pigment identification relies on a subjective assessment of the pigments plausibly in use at a period in time, and the colours likely to be used in areas such as the face or hair, the methodology provides dramatically improved probability of identifying the correct pigment when compared to conventional non-invasive techniques.

We are not aware of any other current analytical technique that could have achieved such an imaging outcome for this painting. The data generated by this study has provided a better understanding of the artist’s technique. The 60 μm spatial resolution allows us to observe with confidence that a majority of the hidden sitter’s face has been achieved as one action. However the disproportionate and blurred form of the ears is indicative of several attempts to achieve the final proportions and features. Degas is reported as having painted “pixie” like ears at about this period^46^. By examining single elemental maps of the painting it is possible to observe such a “pixie” like ear shape (*e.g*., Mn and Fe, Fig. 3) which appears to have been reworked to a more conventional form (*e.g*., Co and Hg, Fig. 3). Careful study of the data reveals numerous intricacies of painting technique and brush stroke direction of the underpainting. It reveals stylistic information and elemental composition information that is unlikely to be reproducible by persons attempting to copy a work, and the technique has strong potential for application in authentication studies^4^,^5^.

Consideration has been given to the properties of synchrotron radiation, and the research group used visible and chemical observation to look for radiation-induced change in preliminary experiments. Pigment binder matrices were studied by Fourier Transform Infrared (FTIR) spectroscopy before and after extended X-ray exposure at the XFM beamline, and spectroscopic changes were not detected. No evidence for any chemical or physical change was observed for radiation doses 10,000 times that reported for this study, which is in accord with recent findings by other research groups using intense radiation sources^47^,^48^.

This study has successfully demonstrated a virtual reconstruction of a hidden portrait by Edgar Degas and has delivered a better understanding of his work and artistic practices. The authors propose that the unfolding technological developments for examining artwork using synchrotron radiation-based techniques will significantly impact the ways cultural heritage is studied for authentication, preservation and scholarly purposes. We anticipate that the high quality outcome presented here and the propagation of the rapid-scanning XRF detector technology used will further stimulate growing interest in the better understanding of our cultural assets. Parallel work using portable XRF systems^7^ is demonstrating that a version of the technique is becoming viable (at substantially reduced spatial resolution and increased data collection time) outside of a synchrotron facility, raising a strong likelihood that precedents being set at synchrotron facilities will directly influence emerging field-based technologies. Until recently XRF large area scanning facilities were built in-house, and this had limited the technique’s availability. With the introduction of commercial large scanning area instruments on the market^49^, the technique has the potential to expand rapidly.

## Methods

### XRF imaging

The scanning XRF mapping of the painting *Portrait of a Woman* was performed at the X-ray fluorescence microscopy (XFM) beamline of the Australian Synchrotron^31^. The X-ray fluorescence was acquired with the Maia 384A detector array, which integrates the sample stage motion with continuous fly scanning, leading to zero data readout overhead^50^,^51^. An incident excitation beam energy of 12.6 keV was used to circumvent intense fluorescence from the Pb L absorption edges, which would originate primarily from the painting’s Pb-based ground layer and thereby limit detection sensitivity to other elements in the pictorial paint layers. The low-energy sensitivity of the detector is limited to approximately 4 keV, thus Pb-M fluorescence (~2.3 keV) was not detectable for example. The energy resolution of the detector is 375 eV at Mn Kα.

The artwork was fitted to a custom manufactured cradle for scanning. The painting was placed approximately 13 mm from Maia detector rather than the optimal distance of 10 mm, since the painting was not perfectly flat. The painting is shown mounted at the XFM beamline in Supplementary Material Fig. S1. A 426 × 267 mm^2^ area was raster-scanned at 16.4 mm s^−1^, providing a dwell time of approximately 3.7 ms per 60 × 60 μm^2^ pixel and yielded a 31.6 megapixel data set in 33 h. Given the 10 × 10 μm^2^ incident beam size used, the average time an area of the painting was in the beam was 0.6 ms. The average incident flux on the painting was 1.5 × 10^9^ photons s^−1^.

Full spectrum XRF data were deconvoluted into elemental maps using the dynamic analysis^52^ method implemented in the GeoPIXE software suite^53^. The highly complex nature of a painting with its varying pigments, paint layers and thicknesses is inherently difficult to model with respect to the calculation of X-ray fluorescence yields. Thus the elemental concentrations are considered semi-quantitative. We have shown the elemental maps as the square root of the observed counts and adjusted the maximum intensity threshold to best represent the high dynamic range data in a single image.

### Raman scattering

An oil-based lead white paint sample (basic lead carbonate, 2PbCO_3_·Pb(OH)_2_) was applied to a commercially prepared canvas support. The XRF spectra of lead white was obtained at 12.6, 12.8 and 13.0 keV incident energies with the Maia detector, each with a dwell time of 25 s.

### Infrared image

The reflected infrared image was acquired with a Sony DSC-V1 digital camera (1/30 s exposure, *f*/2.8 aperture) with its infrared filter removed. The light source was a 150 W Philips infrared heat lamp (Infraphil type KL7500A/90) incident to front of the painting at 1 m distance.

### False colour image reconstruction

A false colour image was created by treating the elemental maps as layers of a single image. Each elemental map was exported as a 32-bit TIFF image of 124 MB size. The custom software constructs an image layer for each elemental map by manual assignment of a single solid colour to the image layer, and each pixel in the image layer is manually assigned an opacity that is proportional to the corresponding measurement in the elemental map. For an elemental map, *E*, the corresponding layer opacity, *L*, is given by *L* = (*αE*^2.2^)^*γ*/2.2^, where α is a transparency level and γ is a gamma correction value that acts like a contrast setting. These parameters help compress the high dynamic range measurements in the elemental maps down to the range visible on computer screens. Finally, all layers are merged by alpha composition using the “over” operator^54^, where the stack of layers are combined from the bottom layer up to the top. All parameters (layer stacking order, layer colour, opacity) are manually selected for each image layer. The software enables processing of the full data set to generate a composite image with the same resolution as the initial data in a processing pipeline fashion that requires only a tiny fraction of the computer memory (compared to a similar operation performed in image editing software) allowing merging of any number and any resolution of coloured elemental maps. Further information about the false colour image reconstruction is given as Supplementary Material and the parameters used for the reconstruction are given in Table S1 and the coloured elemental maps used are presented in Fig. S2.

## Additional Information

**How to cite this article**: Thurrowgood, D. *et al*. A Hidden Portrait by Edgar Degas. *Sci. Rep.* **6**, 29594; doi: 10.1038/srep29594 (2016).

## Supplementary Material

Supplementary Information
