# Supplementary material for: A Hidden Portrait by Edgar Degas
Source: Sci Rep. 2016 Aug 4;6:29594. doi: 10.1038/srep29594 (PMC4973632; doi:10.1038/srep29594)
Supplement: Supplementary Information [file srep29594-s1.pdf]

Supplementary Information

A Hidden Portrait by Edgar Degas

David Thurrowgood[1†*], David Paterson[2], Martin D. de Jonge[2], Robin Kirkham[3], Saul Thurrowgood[4] & Daryl L. Howard[2*]

[1]National Gallery of Victoria, Melbourne, Victoria, Australia. [†]Present address: Queen Victoria Museum and Art Gallery, Launceston, Tasmania, Australia. [2]Australian Synchrotron, Clayton, Victoria, Australia. [3]The Commonwealth Scientific and Industrial Research Organisation, Clayton, Victoria, Australia. [4]Queensland Brain Institute, University of Queensland, Brisbane, Queensland, Australia. Correspondence and requests for materials should be addressed to D.T or D.L.H. (David.Thurrowgood@launceston.tas.gov.au or Daryl.Howard@synchrotron.org.au)

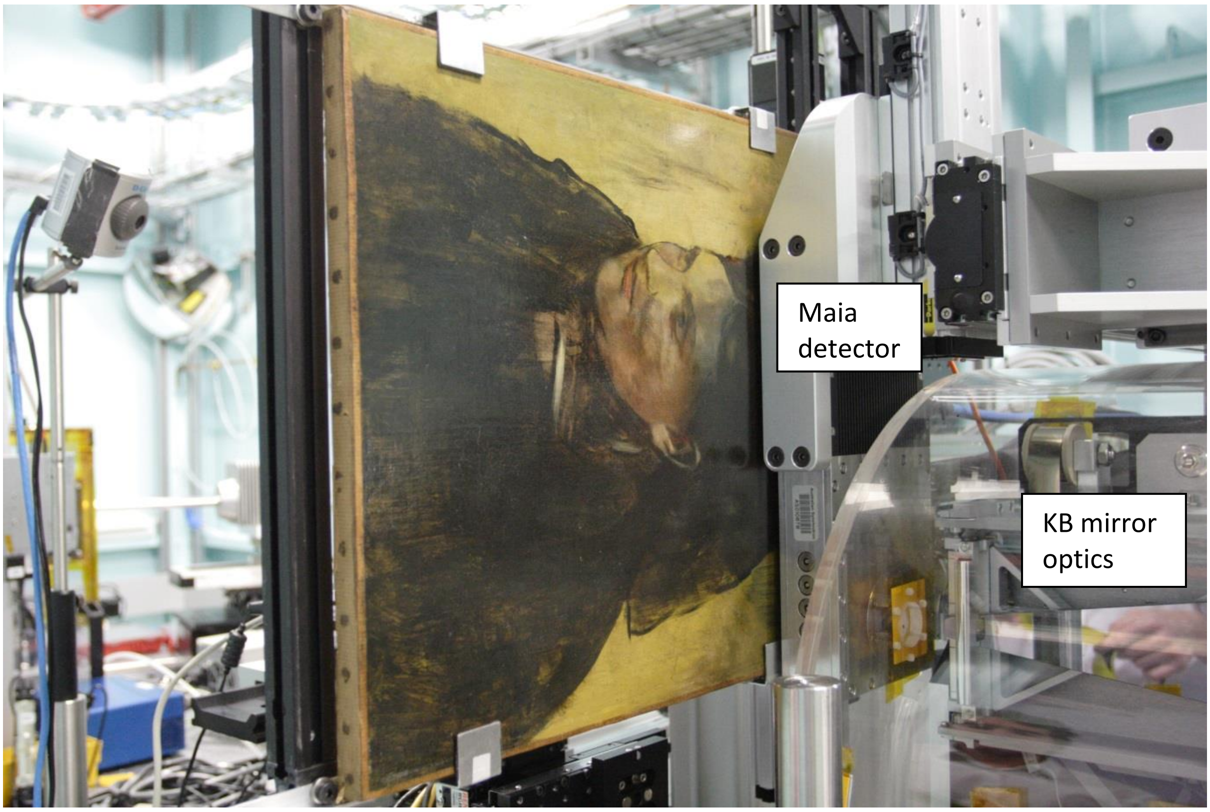

Figure S1 | *Portrait of a Woman* mounted at the XFM beamline. The Maia 384A energy dispersive detector array is positioned in front of the painting with the Kirkpatrick-Baez (KB) mirror focusing optics located upstream. (Edgar Degas, French, 1834-1917, Portrait of a Woman (Portrait de femme) c. 1876-80, oil on canvas, 46.3 × 38.2 cm, National Gallery of Victoria, Melbourne, Felton Bequest, 1937).

False colour image reconstruction. Colour renderings improve the ability to observe and highlight features of interest. The false colour image reconstruction method makes it possible to exaggerate the intensity and highlight location of particular elements, or apply an entirely artificial colour to an element. Thus in the case of *Portrait of a Woman* it is possible to re-process the image to accentuate features from the lower or upper image, or a combination of both. It is possible to use this as a tool to aid the attribution of pigment identity. Where an element that is capable of producing a variety of coloured pigments, separate reconstructions can be made with the colour possibilities, and the resulting reconstruction compared to other works by the artist.

Processing of the data using commercially available software did not yield effective results even when only three elements were overlayed, generally giving mixed shades of brown and poorly defined reconstructed features. The authors are not aware of other systems which enable successful and semi-automated multi-element overlays, while retaining data integrity for large data sets.

For an elemental map, E, the corresponding layer opacity, L, is given by $L = (\alpha E^{2.2})^{\gamma/2.2}$, where α is an transparency level and γ is a gamma correction value that acts like a contrast setting. These parameters help compress the high dynamic range measurements in the elemental maps down to the range visible on computer screens.

Element layers were assigned colour by applying RGB values consistent with the proposed pigment identity. Transparency of the element layer was assigned to achieve a plausible reconstruction, such as, skin tones consistent with other Degas paintings from the period. Elements were chosen to be layers in the colour reconstruction if they were above trace levels, that is, they were likely to add to the visual reconstruction. The colour reconstructed image was not observed to impact if additional trace elements were included as additional layers. The ordering of the layers has an effect on the resulting image. The ordering was chosen subjectively in that it produced an image of similar appearance to other Degas works. For example, applying a high transparency layer over low

transparency layers did not achieve a "good" reconstruction, even though such an image can be useful for interpreting the artist's technique. The selection of a "wrong" colour for a layer (by proposing other pigment identities) gave a poor reconstruction; for example if the sitter had green hair, which was inconsistent with the artist's known practices. Different RGB combinations produce similar results, up to a point. The results are not particularly sensitive to the absolute choice of RGB values, for example, slightly adjusting the RGB values for the blue layer does not result in a significantly different reconstruction.

From the variety of calculated overlay reconstructions we selected the version which most closely matched how an unobscured artwork may have appeared. A limitation of this layering method is that each element can exist at only one location in the layer stack, theoretically reducing the quality of the final reconstruction (real paintings are not defined layers, but mixtures). We would hope to further refine our computational technique.

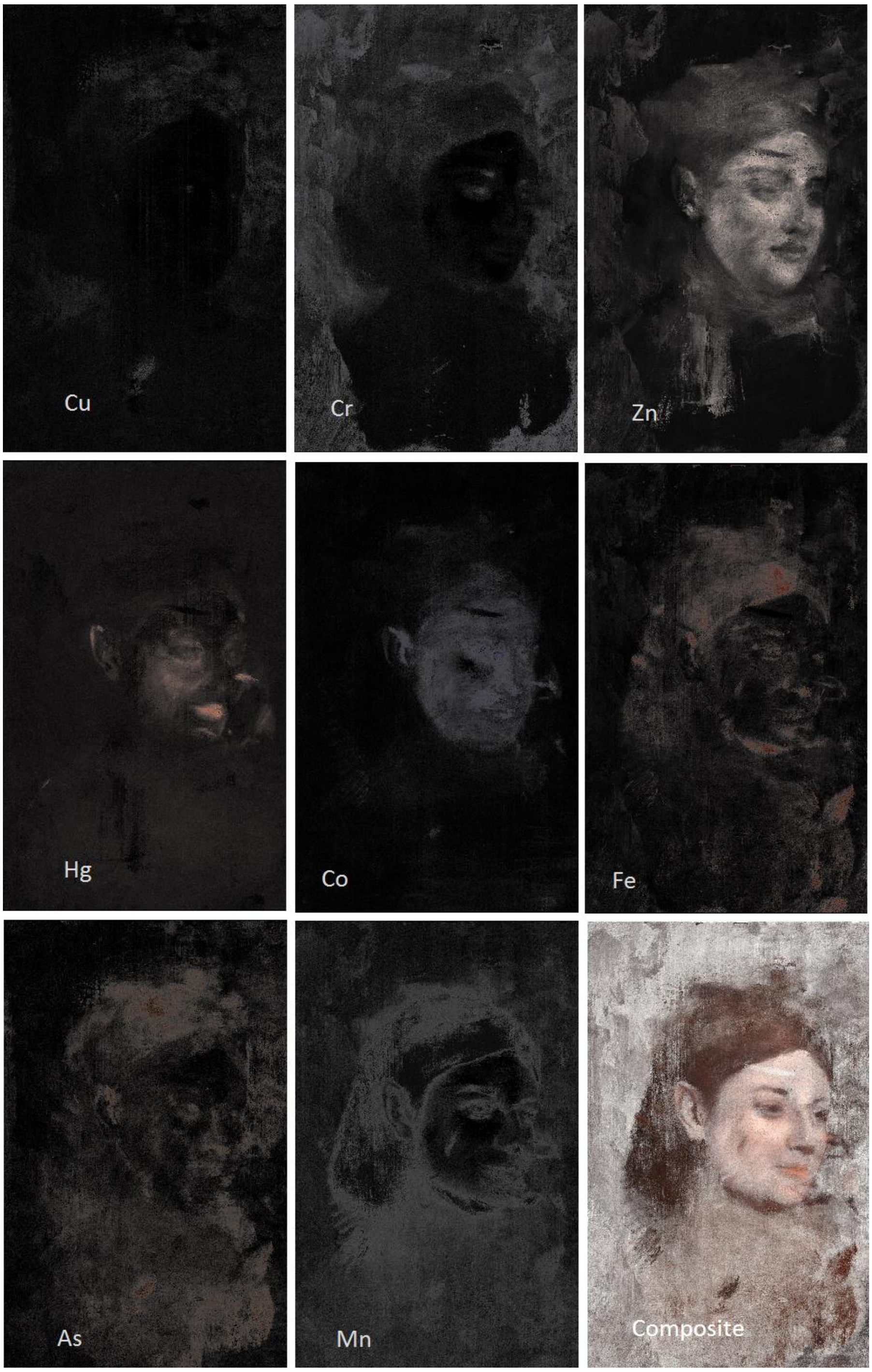

Figure S2 | Assignment of colour to elemental distributions. Documented pigment colours can be assigned to individual elemental distributions. The software overlays the coloured maps to generate a composite false colour image situated on a white base layer . By altering the colour assigned to an elemental distribution is possible to accentuate features, and assess the plausibility of the assigned pigment. For example if Cr was designated orange ($PbCrO_4{\cdot}PbO$) instead of green (Cr_2O_3) the composite image would not resemble other works by the artist at period, and the pigment identity assignment is probably incorrect. The elements, colour assignment and opacity assigned to the elemental maps to produce the composite image published here are given in Table S1. (Edgar Degas, French, 1834-1917, Portrait of a Woman (Portrait de femme) c. 1876-80, oil on canvas, 46.3 × 38.2 cm, National Gallery of Victoria, Melbourne, Felton Bequest, 1937).

Table S1 | False colour image reconstruction parameters used for *Portrait of a Woman*.

Element	Layer number	Colour (R, G, B)	α (transparency)	γ (gamma correction, contrast)
Cu	1 (top)	43, 52, 34	9.2	0.96
Cr	2	25, 30, 24	8.7	0.96
Zn	3	254, 214, 196	$3.1x10^{-5}$	0.47
Hg	4	192, 80, 46	$5.4x10^{-3}$	0.47
Co	5	5, 1, 26	0.64	0.96
Fe	6	59, 12, 0	$1.3x10^{-2}$	1.6
As	7	42, 19, 8	1.6	1.2
Mn	8	0, 0, 0	8.7	1.0
(Background)	9 (bottom)	255, 255, 255	N/A	N/A
